# Influence of breathing state on the accuracy of automated patient positioning in thoracic CT using a 3D camera for body contour detection

**DOI:** 10.1007/s00330-021-08191-3

**Published:** 2021-07-29

**Authors:** Ronald Booij, Marcel van Straten, Andreas Wimmer, Ricardo P. J. Budde

**Affiliations:** 1 Department of Radiology & Nuclear Medicine, Erasmus MC, P.O. Box 2240, 3000 CA Rotterdam, The Netherlands; 2 Computed Tomography Division, Siemens Healthineers, Forchheim, Germany

**Keywords:** Tomography scanners, X-ray computed, Multidetector computed tomography, Thorax

## Abstract

**Objective:**

To assess the influence of breathing state on the accuracy of a 3D camera for body contour detection and patient positioning in thoracic CT.

**Materials and methods:**

Patients who underwent CT of the thorax with both an inspiratory and expiratory scan were prospectively included for analysis of differences in the ideal table height at different breathing states. For a subgroup, an ideal table height suggestion based on 3D camera images at both breathing states was available to assess their influence on patient positioning accuracy. Ideal patient positioning was defined as the table height at which the scanner isocenter coincides with the patient’s isocenter.

**Results:**

The mean (SD) difference of the ideal table height between the inspiratory and the expiratory breathing state among the 64 included patients was 10.6 mm (4.5) (*p* < 0.05). The mean (SD) positioning accuracy, i.e., absolute deviation from the ideal table height, within the subgroup (*n* = 43) was 4.6 mm (7.0) for inspiratory scans and 7.1 mm (7.7) for expiratory scans (*p* < 0.05) when using corresponding 3D camera images. The mean (SD) accuracy was 14.7 mm (7.4) (*p* < 0.05) when using inspiratory camera images on expiratory scans; vice versa, the accuracy was 3.1 mm (9.5) (*p* < 0.05).

**Conclusion:**

A 3D camera allows for accurate and precise patient positioning if the camera image and the subsequent CT scan are acquired in the same breathing state. It is recommended to perform an expiratory planning image when acquiring a thoracic CT scan in both the inspiratory and expiratory breathing state.

**Key Points:**

• *A 3D camera for body contour detection allows for accurate and precise patient positioning if the camera image and the subsequent CT scan are acquired in the same breathing state*.

• *It is recommended to perform an expiratory planning image when acquiring a thoracic CT scan in both the inspiratory and expiratory breathing state*.

## Introduction

Two of the main technological developments in computed tomography (CT) automatic exposure control (AEC) have been automated tube-current modulation (ATCM) and automatic tube voltage selection. These optimize radiation dose while maintaining image quality (IQ) [1]. AEC generally relies on the CT localizer radiograph to determine patient size. Positioning of a patient lower or higher than the scanner isocenter affects the patient’s shape on the localizer radiograph, thereby affecting the AEC behavior [2–4]. Therefore, ideal patient positioning, defined as setting the table height such that the patient’s isocenter coincides with the scanner isocenter, is important. Deviation from the ideal table height can result in an increase of the relative organ dose or a deterioration of the IQ [3, 5]. Besides that, the breathing state of the patient (inspiratory or expiratory) likely results in different anterior-posterior chest sizes and hence in different ideal table heights. Furthermore, clinical indications like e.g. small airway obstruction and cystic fibrosis may call for image acquisition in both full inspiration and expiration to assess the lung parenchyma and to recognize air-trapping [6, 7].

Recent studies described accurate patient positioning with the aid of a commercially available 3D camera [8, 9]. Ideal table height is suggested by the 3D camera with the aid of a single planning image triggered by the radiographer when the patient is lying on the scanner table in the target pose of the examination. The ideal table height for the individual patient and the scheduled examination is proposed such that the isocenter of the body region to be examined and scanner isocenter align. We hypothesized that obtaining the planning image in a breathing state that differs from the breathing state during the scan will result in less accurate patient positioning by the 3D camera. The aims of this study were to [1] determine the difference in ideal table height for a CT scan in inspiration versus expiration and [2] assess the influence of a mismatch in breathing state between the planning image and the actual CT scan on the accuracy of automated patient positioning using a 3D camera.

## Materials and methods

The study was conducted in accordance with the declaration of Helsinki and international standards of Good Clinical Practice. The medical ethics committee of our hospital waived the need for informed consent.

### Study design and patient selection

Two CT scanners at our institution are equipped with a commercially available 3D camera (Siemens Healthineers) for body contour detection: a dual-source CT scanner (SOMATOM Drive; software version VA62A, Siemens Healthineers) and a single-source CT scanner (SOMATOM Edge Plus, software version VB10, Siemens Healthineers). All patients over the age of 18 who underwent a thoracic CT examination on these scanners in inspiration and expiration without contrast enhancement within a period of 8 months (between November 2017 and June 2018) were considered for inclusion. Exclusion criteria were obvious patient movement, repositioning after body contour detection by the 3D camera, blockage of the camera view by large items, and cases in which the field-of-view (FOV) did not cover the patients’ total anterior-posterior extent.

**Fig. 1:**
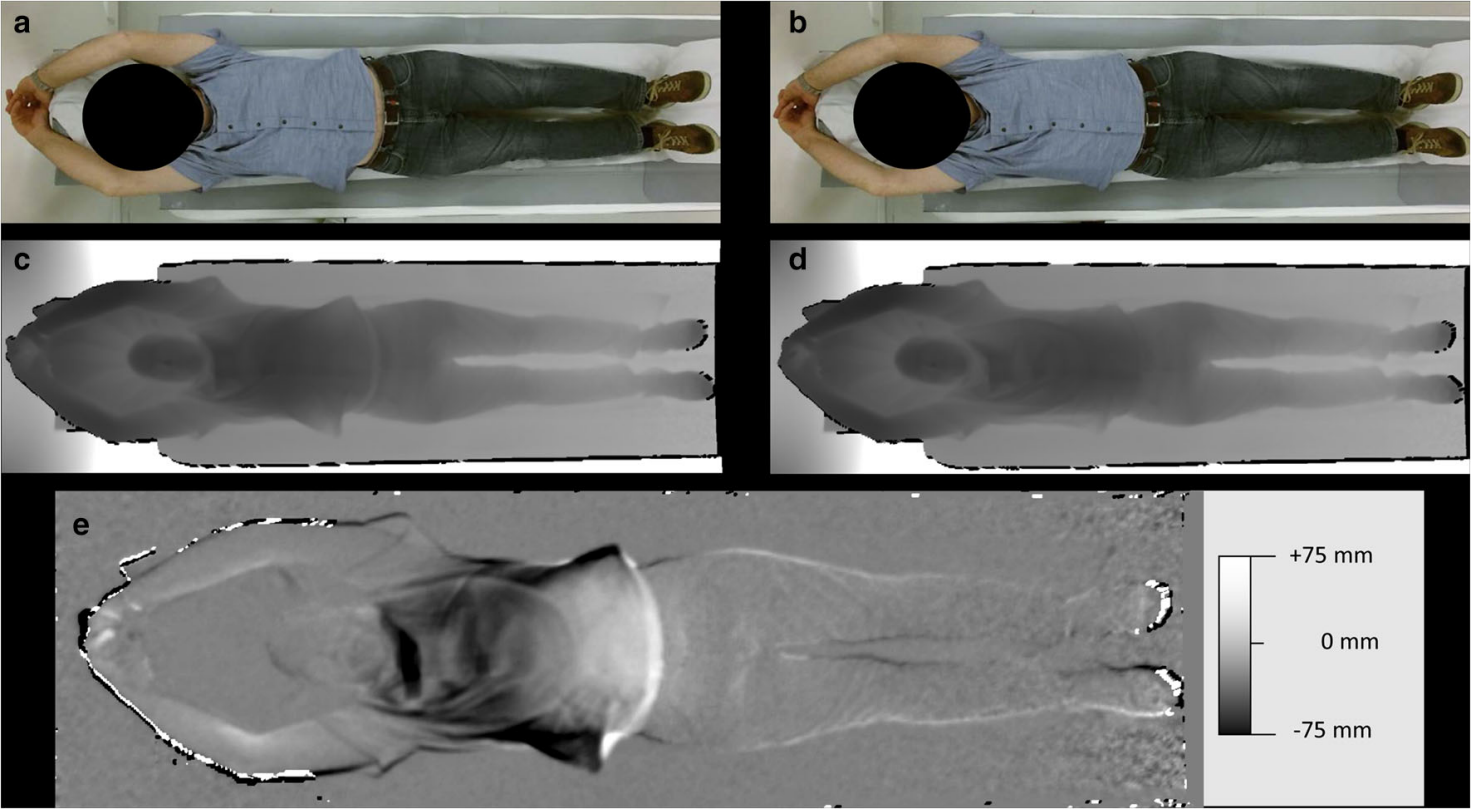
(**a**–**e**) Case presentation of a patient in the inspiratory (**a** and **c**) and an expiratory (**b** and **d**) breathing state. **a**, **b** Color image taken by the 3D camera system. **c**, **d** Depth image corresponding to measured depth values. The depth image is displayed with a gray scale corresponding to measured depth values. The gray level window center is 1.8 m (= the distance from the camera image plane to objects) with a width of 0.88 m to provide the best contrast for the patient. Black denotes regions without measurements. **e** The image represents the difference of inspiration depth image minus expiration depth image and is displayed with a gray level window center of 0 mm and a width of 150 mm to provide the best contrast for the thorax region. In inspiration, the chest is raised (closer to the camera) by 15–25 mm, whereas the abdomen is lowered (away from the camera) by 30–40 mm

For the first part of the study, the difference in ideal table height between an inspiratory and expiratory breathing state was determined by including all patients with an inspiratory and an expiratory CT scan. For the second part of the study, a subgroup with only patients with both the expiratory and inspiratory 3D camera images and accompanying CT scans was considered for inclusion (Fig. 1). Before the first CT scan was performed, 3D camera planning images were taken by asking the patients within the subgroup to perform a deep expiration and a deep inspiration, respectively. CT images were reconstructed at a slice thickness of 3.0 mm and an increment of 3.0 mm.

### Patient positioning using a 3D camera for body contour detection

The 3D camera is part of the CT system and attached to the ceiling, facing down onto the patient table. The camera acquires a color and a depth image. Each pixel in the depth image describes the distance from the camera to the closest object surface. The image analysis starts after taking the planning image. Then, the algorithm detects the patient and estimates the body contour of the patient using the depth measurements and the known table position and shape. The 3D camera proposes the ideal table height for the individual patient and the scheduled examination such that the isocenter of the selected scan range and the scanner isocenter align. Therefore, a virtual patient avatar is fitted to the camera data. The avatar is a statistical shape model, which in the fitting process assumes the pose, and body proportions of the patient found in the depth data. The isocenter curve of the avatar is finally averaged across all slices of the body region selected. The 3D camera algorithm applied is equivalent to that described in detail before in previous studies [8, 10].

### Calculation of patient positioning accuracy

Skin surface was extracted from the CT data in each axial slice. The results of the skin extraction were used to calculate the patients’ centerpoint in the axial plane, which were averaged over all slices along the z-axis to assess the patient isocenter. These parameters are required to determine the ideal table height as described in detail before [8].

In the clinical workflow, table height suggested by the camera is based on the latest planning image only. Therefore, analysis of proposed patient positioning based on the inspiratory and expiratory camera image was assessed offline. Offline system performance reflects the real-world situation, as no data or user input other than obtained in the clinical routine was used offline. Accuracy in patient positioning is regarded as the deviation of the table height proposed by the camera algorithm from ideal table height, expressed as a single and absolute value in millimeters.

### Statistical analysis

Descriptive statistics were calculated for the differences between ideal table height for an inspiratory and expiratory scan as well as corresponding 3D camera images for the subgroup in which they were available. Normality of data was tested with the Shapiro-Wilk test. A paired *T*-test was applied to assess statistically significant differences of the ideal table height deviation between the inspiratory and expiratory breathing state. Statistical analysis was performed using SPSS (version 25, IBM Corp). A *p* value < 0.05 was considered statistically significant.

## Results

After exclusion of three cases from analysis due to the anterior-posterior extent of the patient not being fully included in the FOV, sixty-four CT studies with both an inspiratory and an expiratory CT scan were available for analysis of differences between the ideal table height for CT scans in inspiration and expiration. For the subgroup, 43 patients of the 64 patients, ideal table height suggestion by the 3D camera was obtained by offline analysis for both breathing states. No patients had to be excluded due to obvious movement, repositioning, or blockage of the camera view.

### Ideal table height as a function of breathing state

**Fig. 2:**
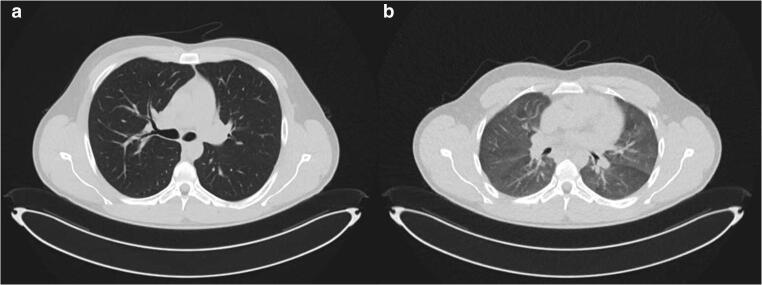
(**a**, **b**) Adult patient with an inspiratory (**a**) and expiratory (**b**) breathing state. **a** Axial image of the thorax in an inspiratory breathing state. **b** Axial image of the thorax in an expiratory breathing state. Images represent the same slice location and the same display window: window width 1500 HU and window center −400 HU

Figure 2 illustrates an example of an inspiratory and expiratory breathing state. Among all 64 patients, the mean (SD) difference for the ideal table height between inspiration and expiration was 10.6 mm (4.5) (*p* < 0.05). The maximum and minimum absolute difference of the ideal table height between the inspiratory and expiratory scan was 24.3 mm and 2.6 mm, respectively. In all cases, the ideal scanner table height position was lower for CT scans in inspiration than for those in expiration.

### Patient positioning accuracy of a 3D camera for inspiratory and expiratory thoracic CT

When considering the same breathing state for both the CT scan and the planning image, the mean (SD) difference between the ideal table height and the table height proposed by the 3D camera was 4.6 mm (7.0) and 7.1 mm (7.7) for the inspiration and expiration breathing state, respectively (*p* < 0.05).

**Fig. 3:**
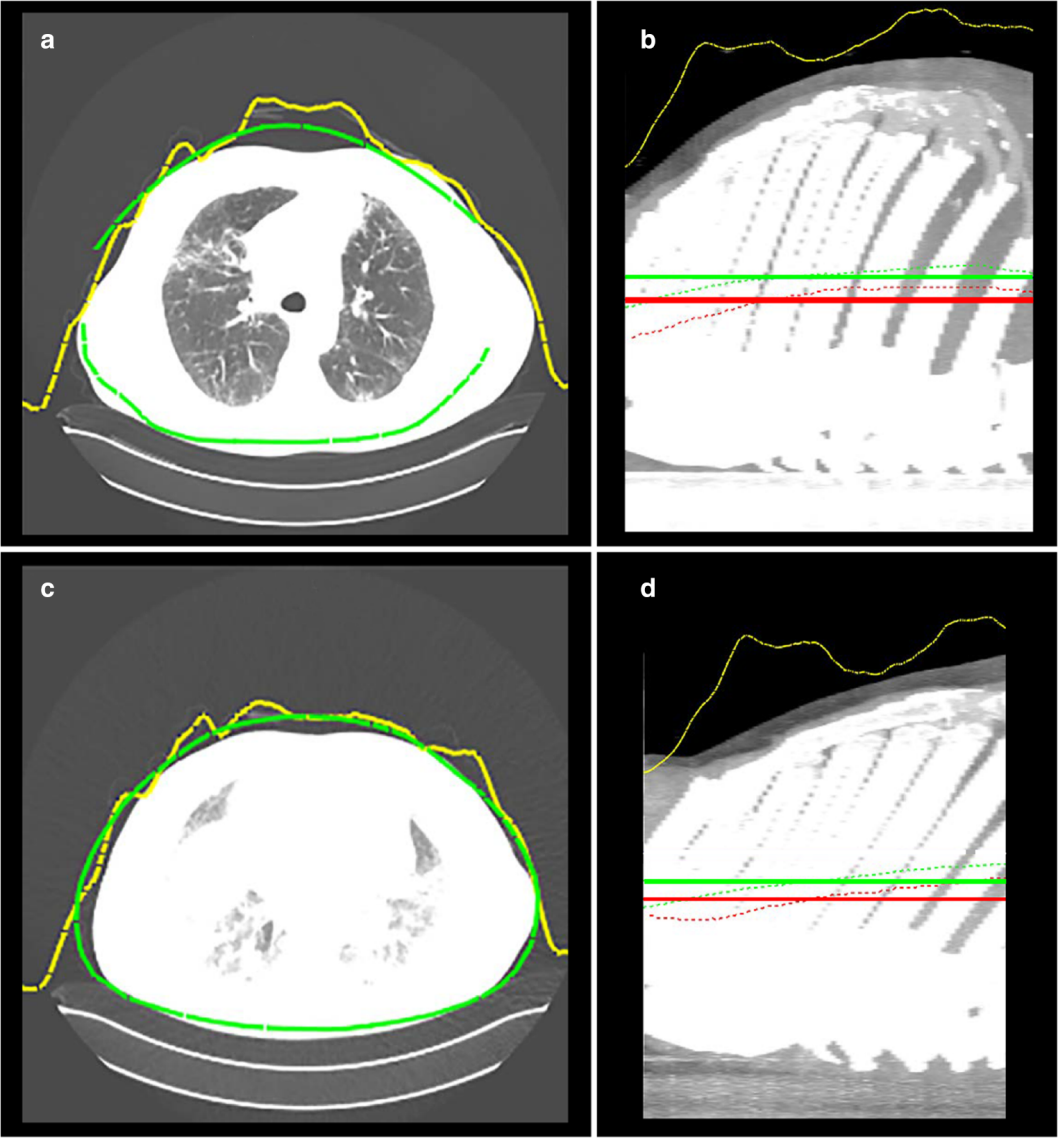
(**a**–**d**) Case presentation of a patient with an inspiratory (**a** and **b**) and expiratory (**c** and **d**) breathing state with depth measurements and the patient isocenter. **a**, **c** Axial image of the thorax with depth measurements (yellow line) by the 3D camera and the body contour (green) estimated by the algorithm. **b**, **d** Sagittal image of the thorax with patient positioning accuracy: green horizontal line: average patient isocenter estimated by the camera, green dotted line: avatar isocenter curve, red horizontal line: average patient isocenter (ideal table height), red dotted line: patient isocenter per axial cross-section, yellow line: depth measurements

The accuracy in patient positioning was lower if an inspiratory 3D camera planning image was used for an expiratory CT scan with a difference of 14.7 mm (7.4) to the ideal position (*p* < 0.05). Figure 3 demonstrates the differences between the inspiratory and expiratory breathing states, corresponding depth measurements, and the corresponding patient isocenters.

## Discussion

In this study, we assessed the influence of breathing state on the differences in ideal table height and on the accuracy of automated patient positioning using a 3D camera. In the first part of our study, different ideal table heights were observed between an inspiratory and an expiratory breathing state CT scan. Previously, we reported on the accuracy of automated patient positioning, where we assumed that the camera image and the subsequent CT scan were acquired in the same breathing state [8, 11]. Using a different breathing state for the planning image and the subsequent CT scan may result in less accurate patient positioning by the 3D camera, as demonstrated in Table 1.

**Table 1:** 3D camera mean patient positioning accuracy and standard deviation (SD) for all combinations of breathing states while taking the camera image and performing the subsequent CT scan

Absolute table height deviation from ideal table height as a function of breathing states		
	Breathing state CT scan	
Breathing state 3D planning image	Inspiratory	Expiratory
Inspiratory	4.6 (7.0)	14.7 (7.4)
	*< 0.05*	*< 0.05*
Expiratory	3.1 (9.5)	7.1 (7.7)
	*< 0.05*	*< 0.05*

Data are mean (SD) in mm with *p* value

Interestingly, the difference in table height between an expiratory planning image and the inspiratory CT scan was slightly smaller than the difference between an inspiratory planning image and the inspiratory CT scan. However, the SD, i.e., imprecision, was higher when an expiratory planning image was combined with the inspiratory CT scan (Table 1). In the end, a better precision is preferred over a slightly better accuracy and we recommend making an inspiratory planning image for an inspiratory scan. In case both an inspiratory scan and an expiratory scan are made, an expiratory planning image is preferred because of the much better accuracy for the expiratory scan. Further research is needed to look into other solutions which do not demand a change in workflow, e.g., possibilities to adapt the table height in between the CT scans for different breathing states.

The effect of improper patient positioning on radiation dose and image quality was not investigated in this paper, since the influence of off-centering patients in a CT scanner on IQ and radiation dose has been extensively described in several publications [2, 12, 13] and one also on dose saving with the aid of a 3D camera equivalent to the one described in this study [14]. For instance, Monte Carlo simulations in a phantom study resulted in relative organ dose differences of the lungs and heart between 5 and 12% when a patient was positioned 10–20 mm off-center [3]. When interpolating this data to the maximum absolute table height deviation of 14.7 mm (Table 1), the associated dose change would be approximately 8%. Additionally, vertical off-centering may influence the CT number, expressed in Hounsfield units (HU) [13] and organ dose can be higher [3]. In our pursuit of optimization, it remains important to do everything possible to achieve optimal scanning protocols. The difference between the ideal table height of inspiration and expiration was small. Even if individual optimization steps seem small, their combined effect adds up. To optimize radiation dose and IQ, it is essential to strive for proper patient positioning and thereby improved operation of bowtie filtering and ATCM [15].

There are limitations to this study that require considerations. The calculation of the isocenter for both the expiratory and inspiratory 3D camera images and accompanying CT scans was based on the actual scan range. In routine operations, the algorithm uses the scan range that is defined on the planning image (= color photograph taken by the camera) prior to obtaining the localizer radiograph and scanning the patient. Consequently, this suggested table height may differ from the suggested table height based on the scan range defined on the planning image. Considering the small patient cohort and after performing a post hoc power analysis, we decided it was more appropriate to present the data when considering all patients (both female and male) and not to perform a subgroup analysis for gender. Additionally, no analyses were performed for different chest shapes.

In conclusion, a 3D camera allows for accurate and precise patient positioning if the camera image and the subsequent CT scan are acquired in the same breathing state. Therefore, we recommend to acquire the 3D camera image and subsequent CT scan in the same breathing state if only one CT scan of the thorax is needed (either in inspiration or in expiration): a 3D image in inspiration for a CT scan in inspiration and a 3D image in expiration for a CT scan in expiration. If both the inspiratory and expiratory breathing state thoracic CT scans are required, it is recommended to perform the 3D camera image in expiration.

## Abbreviations

AEC: Automatic exposure control
ATCM: Automated tube-current modulation
CT: Computed tomography
FOV: Field-of-view
HU: Hounsfield units
IQ: Image quality
SD: Standard deviation

## Declarations

### Guarantor

The scientific guarantor of this publication is Ricardo Budde.

### Statistics and biometry

One of the authors has significant statistical expertise.

### Methodology

• Prospective

• Experimental

• Performed at one institution
